# Maintaining the future through recurrent crises

**DOI:** 10.1177/1354067X231204298

**Published:** 2023-10-12

**Authors:** Oliver Clifford Pedersen

**Affiliations:** 27214 Institut de psychologie et éducation, University of Neuchâtel and nccr – on the move, Neuchatel, Switzerland

**Keywords:** Crisis, future, im/mobility, imagination, temporality

## Abstract

Einar, a 94-year-old Faroese man who has always resided on the island of Suðuroy, has lived through several societal crises. In this article, I explore his experiences of living through three of them, highlighting his ability to maintain an imagination of in the future and detailing how crises interact over time. I propose that Einar’s upbringing in the cycles of crisis characterising fishery, combined with other factors, led him to develop a particular engagement with the future that mitigated crisis-induced uncertainties. Previous crises served as resources to address current calamities, as such experiences taught Einar that, in even the direst situations, the future will eventually improve. However, Einar temporarily sight of the future when the Faroese fishery industry imploded in the 1990s. Einar questioned whether sufficient resources existed to allow him and the village to recover. The 1990s crisis momentarily gained a personal character; however, due to his age, stable socioeconomic position, and close social and affective ties to the people on Suðuroy, he reaffirmed his decision to stay on the island. Einar’s story showcases the importance of considering people’s experiences and unique positions amidst societal crises—be they temporary, slow, recurrent, or chronic, but also of studying experiences of time. Numerous factors constitute the membrane regulating when societal crises become personal. Crises cannot be understood as singular or isolated events. Instead, crises must be comprehended through their cumulations and entanglements, which involve people’s imagination of the future in unpredictable ways.

## Introduction

Einar is a 94-year-old Faroese man who has lived through several societal crises. These are periods that “expose the seams of temporality” (Knight & Stewart, 2016, p. 3) and constitute times in which imaginations of the future are characterised as uncertain (Kleist & Jansen, 2016), contested, and nebulous. Einar grew up on the Faroese island of Suðuroy back when the island was one of the socioeconomic and technological centres of the Faroes in the 1920s and 1930s (Holm & Mortensen, 2002; Joensen, 1987). Foreign anglers docked with their motorised smacks, new hydroelectric and ice plants were constructed, ideas of unionisation took hold, and British warships and up to 8,000 soldiers created a peaceful protectorate during the Second World War (Sølvará, 2020). These myriad im/mobilities and emerging infrastructural projects instilled a temporal logic of progress (Larkin, 2013), and the encounters engendered Einar’s will to stay despite imperatives to develop through mobility (Pedersen & Zittoun, 2022). In this continuation of Einar’s story, I explore his imagination of the future through three societal crises, revealing how disparate crises resonate, and discuss questions concerning temporality more broadly.

As detailed elsewhere (Pedersen, 2022), my colleague, Emmanuel Charmillot, and I met Einar at a reception honouring a retiring hospital director. An acquaintance unexpectedly invited us to accompany her to the event, and one of her colleagues kindly offered us a ride to the now industrialised harbour front. The reception was held on the first floor of an old sail-making place. Upon ascending the stairs, the retiring hospital director warmly greeted us at the landing. The room had white walls and exposed wooden beams. A table with a lavish selection of finger foods and drinks was set at the far end. Amidst speeches and short musical acts, our mutual friend introduced us to Einar. He was seated at the edge of the crowd, near a dessert table laden with coffee and miniature cakes. Our initial conversation with Einar was in English, as neither Emmanuel nor I spoke Faroese. Although Einar spoke English effortlessly, he occasionally switched to Danish after realised that I was Danish. We agreed to meet the subsequent week. Since then, I have had several interviews and conversations with him, most of which lasted hours. Several of these discussions took place in the comfort of his living room, and we also kept in touch over the phone after I departed.

I recall being surprised during one of our conversations in Einar’s living room. The walls were decorated with faded photos and landscape paintings. His couch area was directly in front of a large window overlooking the fjord and village, with the television and an old stereo tower angled to the side. Einar served Faroese beer although he drank tee. His descriptions of living through what had been both categorised and politicised as several crises (Roitman, 2014) did not match my admittedly naive preconceptions. This surprise led me to interrogate the assumption of time inherent in the model of the sociocultural psychological imagination, particularly the assumptions grounding time in sociocultural, material, and geographical conditions (Marková & Novaes, 2020; Valsiner, 2014). Einar described these crises as neither unimaginable nor necessarily as ruptures that rendered the future uncertain. Crises were an intrinsic element of his life and therefore perfectly imaginable. There is no universal experience of crises (Fassin & Honneth, 2022), and in Einar’s case, previous crises and their past and imagined repetition had become a resource for maintaining the future in periods often characterised by high uncertainty. Crises are often defined as an extraordinary and temporally delineated event signalling the collapse of the psychological and societal status quo, which calls for a decision (Koselleck & Richter, 2006). Temporal expectations become obsolete, giving way to a liminality squeezed in-between “two states of normality” (Knight & Stewart, 2016). However, Vigh and others (Mbembe & Roitman, 1995) argued that crises are not exceptional states for those “chronically ill, the structurally violated, socially marginalised and poor” (2008, p. 7). Crisis can be endemic rather than episodic. Vigh qualifies that “wars do not start with the first shot or end with the last” (2008; 9) and crises do not have to be either an event or a process (Bergman-Rosamond et al., 2022; Rydstrom, 2022). The catastrophic can become ordinary (Zhukova, 2022) and slow-building violence can become catastrophic (Nixon, 2011), the climate emergency being an obvious example of the latter. Crises are defined in relation to what was or how matters stand elsewhere, but their imagined qualities are also of considerable importance. Vigh approached crisis as *the* context that gives rise to meanings and actions, challenging the common temporalisation of crisis to also account for “pervasive states”. Crises can have many causes, emerge slowly, cumulate, entangle, an even be productive. Research has indicated how the entanglements of various events come to constitute crises (Rydstrom, 2022); therefore, I instead explore how past and future crises resonate with present ones, informing how people imagine the future. Crises always manifest differently according to people’s circumstances—occasionally in material objects such as electronic equipment (Vigh, 2008) or empty houses (Dzenovska, 2020). This is also the case with Einar.

In the Global North, stable social structures, established institutions, and vast power differentials pervade the common, privileged understandings of crises as temporary events and can therefore cloud conditions of chronic crisis—as Scheper-Hughes also proposed (2008, p. 37). This blind spot might partly explain why I was initially surprised by the way in which Einar narrated his experience of crises because it did not correspond with my assumptions. If crises have no clear temporal quality but can also be endemic, slow, or recurrent, then they might not demand decisions, as Koselleck & Richter (2006) proposed. Instead, researchers need to explore the experiences, imaginations, and possibilities that are destroyed, created, or maintained for different people under specific conditions. While Roitman (2014) warned that crisis is an easy placeholder and discussions concerning what a crisis *does* remain rare, it is important to study how people act in times collectively defined as such. Using crises as an entry point raises the question of whether the concept of irreversible time fully captures how people develop and make sense of the world.

Within sociocultural psychology, the process of imagination is theorised as unfolding in the irreversible flow of time (Zittoun & Gillespie, 2016) while allowing people to “travel in time” imaginatively. However, this conceptualisation risks decontextualising the experience and construction of time, which holds implications for the experience of crises (Bergman-Rosamond et al., 2022). For example, if time has a circular quality, then crises might not represent a rupture ridden with uncertainties. Anthropologists claim that multiple temporalities exist (Adam, 1995) in their relations (Ssorin-Chaikov, 2017), which can “bend” and shape people’s experience of the flow of time (Ringel, 2016). Without dismissing the philosophical premise that time flows in one direction, it is analytically useful to differentiate time from temporality to better account for the situatedness of the imagination (Pedersen, 2022). Integrating temporality as an analytical category of the sociocultural psychological canon prompts questions concerning how people’s imagined futures transform, if at all, in times of crises, but also how the flow in Heraclitus’ river is experienced.

It is useful to distinguish an “external” diagnosis of a societal crisis from how or what a person experiences as a crisis. This distinction mirrors that between the third- and first-person perspectives (Zittoun et al., 2023). However, no one-to-one translation exists and, without distinguishing between the levels of analysis, researchers risk ignoring inequalities that remain hidden when generalising the experience of crisis (Fassin, 2022). I want to account for what is experienced as a crisis and the time in which a crisis manifests because a crisis is not a “monolithic or a momentary experience” (Vigh, 2022, p. 523). In addition to disrupting the future, crises can also bracket the future due to expectations of their recurrence. This approach echoes Zittoun’s paired notions of rupture and transition (Zittoun, 2006). She proposed that both endogenous and/or exogenous events (such as crises) can rupture people’s taken-for-granted experiences, triggering a transition towards a new equilibrium depending on the magnitude (Zittoun, 2006). Nevertheless, Einar’s case was not adequately captured by this perspective because it was the absence of apparent ruptures amidst societal crises that sparked my curiosity.

Over the coming pages, I demonstrate that crises cannot be assumed to destabilise the imagined futures that guides people. I first elaborate on the articulation between temporality and imagination during crises before proposing four factors underlying Einar’s ability to imagine the future. I end by discussing several implications for both the sociocultural psychological study and conceptualisation of the imagination and temporality.

## Temporalities and the imagination during crises

Whereas social crisis relates to dynamics within political, economical and social processes, personal crisis is associated with a state of being, defined by the experience of an aggravated limitation of agency, a truncation of horizons and opacity of futures and possibilities. (Vigh, 2008, p. 13)

I take inspiration from anthropology because the connections between temporalities and imaginings of the future in times of crisis remain sparsely studied within sociocultural psychology because the process is assumed to unfold in irreversible time. At one end of a continuum, crises impoverish or outright eradicate people’s ability to imagine a future. At the other end, crises are fertile breeding grounds for new imaginations. I define imagination as a dynamic social and psychological process nourished by different symbolic resources, guiding both ontogenesis (Zittoun, 2013) and sociogenesis (Hawlina et al., 2020). Imagination is a process (Zittoun et al., 2020), constituted by and constitutive of the sociocultural environment (Zittoun & Gillespie, 2016). However, the role of temporalities remains underexplored (Pedersen, 2022), despite time and space being theoretically linked (Valsiner, 2014). Sociocultural psychological theorisations of the imagination situate the process in the irreversible flow of time, proposing that the imagination enables an escape from the shackles of the present (Zittoun & Gillespie, 2016). Such an approach overlooks the consideration that temporalities are not found but socially and culturally constructed (Adam, 1995) and embedded in power relations (Bhatia & Canning, 2021). Building on Bergson’s processual thinking, Adam (1995) differentiates *events in time* from *time in events*. The former follows the idea of irreversibility (e.g., things happen in flow of time) whereas the latter claims that events are constitute of temporality. Such distinction challenges time as an “unvarying medium through which lives are lived and events unfold” (Neale, 2021). However, people do not experience this flow in a uniform way and their imagination changes depending their unique circumstances. Temporalities can be “subjective, relational, embodied and context dependent” (Neale, 2021, p. 81). This is not an attempt to reject ideas of irreversible time, but rather emphasise a need to consider how temporalities are experienced and constructed to better grasp the process of imagination.

Anthropologists have studied the imagination and temporality in times of crises. Knight and Stewart suggested that crises transform or disrupt experiences of temporality, remarking that: “In moments of extreme crisis, time becomes elastic—the time waiting for a bomb to explode or a fist to land can seem like an eternity, or a blink of the eye” (2016, p. 3). Bryant (2016) illustrated how people experience protracted crisis as an “uncanny” present in the case of Cyprus, with the future nowhere within sight. Griffith made a similar point by revealing how rejected asylum-seekers in Britain kept in indefinite detention experience an enforced uncertainty as a “directionless stasis” outside “normal temporality”, entirely challenging their ability to imagine the future (2013, 2014). While these are clearly different examples, they nonetheless demonstrate that what counts as a crisis for one person might not for the next and emphasise that temporalities matter. Crises offers a good moment to study their seams. Institutions can use time as a tactic of governance (Eule et al., 2019). Knight (2016) proposed that neoliberal austerity measures imposed in Greece led to imaginations of the future that varied between generations.

People can experience a sense of endless waiting for a promised future, which might be in the past (Ferguson, 1999) and haunt present imaginings (Zeitlyn, 2015). Geopolitical events such as deindustrialisation or the introduction of free movement also produced localised crises as people and capital left rural places and smaller towns. Ringel (2018) revealed how shrinkage in an East German town led its inhabitants to engage in continuous efforts to endure—to stretch the present a little longer into the future (Dzenovska & Aistara, 2014). Dzenovska (2020) identified a comparable process in rural Latvia, where residents struggle to maintain the present in the face of material emptiness. She describes the residents as trapped between what once was and what has yet to be. I prefer to use the term “emptying” because it not only shifts the perspective towards processes and exemplifies how states and institutions govern the future (Pedersen, 2022) but also indicates that people imagine the future differently in different circumstances. Emptying explicitly connects temporalities with space, highlighting that experiences of time are embedded in local contexts and always relational, but also how possibilities and imagination are intrinsically linked (Zittoun et al., 2020). People’s life courses are inevitably impacted by temporalities (Neale, 2021)

When crises materialise, people might feel stuck as a result (Hage, 2009)–detached from the linearity and progress that dominates much of contemporary life. Crises can relocate the *elsewhen* in space because it is lost temporally (Ringel, 2023). Studies suggest that mobility is often used to create a more attractive future “at home” for oneself or for relatives (Kleist, 2018; Schielke, 2020). Crises not only impoverish people’s imaginations but can also be impetuses for social movements that promote new futures (Hawlina et al., 2020; Power, 2020). The two extremes of the continuum illustrate that crises can both annihilate people’s imagination of the future, leaving them stuck in a perpetual and hopeless present, but also act as powerful breeding grounds for new imaginations. Hage (2009) proposition that “waiting out” a crisis can represent an active and defiant stance against an ostensible loss of agency lies somewhere between these poles.

I now proceed to explore how Einar maintained the future through two of three societal crises. I propose that several factors aided him in this endeavour: learned presentism, affective ties, stable socioeconomic conditions, and growing up in a sociocultural landscape structured around different temporalities containing in-built crises.

## Temporalities on and beyond Suðuroy

The relationship between the future and crises must be historicised. I therefore begin the story with a return to Suðuroy during Einar’s youth in the 1930s and 1940s. All citations in what follows stem from a few biographical interviews I conducted with Einar, unless otherwise indicated. These interviews were conducted as part of the *nccr – on the move*^1^ research project, which explored questions related to imagination and im/mobilities in small localities. I visited the Faroe Islands five times over a year, often accompanied by my colleague, Emmanuel Charmillot. We experienced both the magnificently lush summer with its white nights and the harsh, dark winters.

Back when Einar grew up, life revolved around two main temporalities that shaped his imagination of the future through subsequent crises. Einar described how social life was intimately linked to the mobility of fish and anglers, responding to seasonal changes:

When the husband was home again between the trips, the fishery trips that is, if it was during the summer, then he should attempt to get as much hay gathered to feed the cows and perhaps a couple of sheep, and then he would leave again, and it was the wife that took over. If the husband was home during spring, and he should fish up around Iceland, and then arrive home when the ship was full, half full, don’t matter, it should last about 1 month to 6 weeks, thereabout. And salted the fish so the fish could keep without ice and that. Then there was time for him placing the potatoes in the earth for the next years. That was how it functioned.

Einar never described himself as much of an angler, suffering from terrible seasickness, but he always felt connected to the industry. He explained how social life and people’s im/mobilities reset each year, a description which also features in historical accounts (Joensen, 1987) and Faroese literature (Brú, 2011). Joensen writes that Faroese anglers usually left with the arrival of spring and its more reliable weather around March 1st. The anglers would then return for two weeks at the end of May or the beginning of June before remaining in the North Atlantic until the end of September (1987, p. 93). Whenever the anglers were back on land, the fields needed to be prepared for either producing fodder so the animals could survive a harsh winter or to produce seeds for next year’s potato harvest. Living in resonance with the seasons informed Einar’s imagination of the future, providing clear expectations of what was to come and how to maintain cyclical temporality by performing the same tasks year after year. Even in the face of societal crises, this temporality offered a degree of certainty concerning an orientation towards the immediate future. Einar and his peers needed only to focus on the tasks at hand. Einar further elaborated on the rhythm of life and im/mobility.

The winter period was for drying, but it was like the men in the villages, they were part of different communities, big and small, which meant that they could help each other. If one or two men were sick, and a boat didn’t sail out because it lacked manning, then people received fish from those who had sailed out. That function penetrated the entire society, both in big, and here if we call ourselves for the big society […] That was simply the system, and if we were so lucky that a grind catch came to the island, from 50 to 200 [whales], then there was a communal distribution system. If the men were out fishing, then people helped each other, even the families, which were not represented, got its share because the neighbour or the family, they helped each other getting their share; fish to one month to two months ahead. People helped each other with many different things, and it was that we could almost call it shared consumption, a shared village.

Such tasks were important in surviving a harsh environment, and the community developed an egalitarian attitude that fashioned a safety net (Gaini, 2013). Egalitarianism ensured that nobody was left without (Gaffin, 1996), which the communal distribution of whale meat attested to. Although the importance of whale meat as a means of subsistence had declined significantly, practically everyone received a share, including those absent from the hunt and killing.

With little else than the sea to turn to, Faroese society developed a path-dependence on fishery (Holm & Mortensen, 2002; Justinussen, 1997), creating structural susceptibilities for economic upswings and downswings. This dependency defined another temporality containing elements of both linearity and cyclicity. People stated that the difference between a *good* and a *bad* year depended on natural and geopolitical factors such as fish prices and stocks, the changing international regulations, and the cost of oil. This temporality is simultaneously characterised by certainties and uncertainties—certainty that a downswing is inevitable (as well as an upswing) but uncertainty as to when it will happen. In contrast to a cyclicality temporality that followed a rigid temporal sequence, the global economic system is opaque and unpredictable. These temporalities are relational in that the former ensured a sense of continuity, whereas the latter maintained a sense of progress. Einar’s imagination of the longer-term future was often ridden with uncertainty and rarely primary or concrete—yet tended to be hopeful. He explained how the oscillation between good and bad years was integral to life on Suðuroy and suggested that the shift from bad to good was often linked to “progress” or “modernisation”, which embodies a linear logic. Crises were common, and the promise inherent in this temporality allowed Einar to maintain an imagination of the future even when its immediate foundation was crumbling. These temporal experiences functioned as a resource for Einar. When present crises swept the country, earlier crises allowed Einar to imagine them as temporary and followed by progress, allowing him to keep crisis-induced uncertainty at bay (Kleist & Jansen, 2016), even during significant exoduses (Guttesen, 1996; Holm, 2007). He just had to weather the storm and focus on the tasks before him. Einar described this as a natural part of a fishery-based society and in his experiences, years of economic hardship produced by adverse fishing condition always turned around. In other words, Einar’s crises did not necessary disrupt temporality and obfuscate the future. Instead, they were experienced as familiar bumps in the road. Crises do not per default break temporalities but can be an anticipated part of them, shaping people’s experiences and imagination.

Altogether, Einar’s life was shaped by two main temporalities, including one in which crises were normalised, which supported his ability to imagine the future through societal crises. The first temporality generated an imagination focused on the short-term future and provided clear steps to actualise it, whereas the second temporality simultaneously engendered uncertain and certain imaginations of the longer-term future that were infused with ideas of progress. Past crises and temporalities guided Einar’s imagination. Identifying the temporalities and situating Einar’s imagination is crucial for understanding how he engaged with the future when external conditions were increasingly dire. Moreover, egalitarian values reduced despair. Egalitarianism guaranteed that, in the most extreme depths of societal crises, people could rely on the community. Einar’s temporal experiences, combined with his exposure to societal downswings that were cushioned by social practices, shaped his imagination. Upswings always followed downturns. This temporality allowed Einar to maintain an imagination of the future even during societal crises. He drew upon past crises to understand current ones, imagining a future beyond them. This created a form of temporal resonance between the various crises.

## Affective relations and the future

Einar’s imagination did not materialise in a social vacuum but always in connection to social others and affective ties (Vygotsky, 2004). Social others bolstered his ability to wait out crises. Research has documented how social and affective ties are significant for how and why people move or remain still (Cole & Groes, 2017; Schapendonk, 2020, p. 20), but studies on imagination have not studied such affects in detail. Einar’s affective ties shaped his imagination of the future, consolidating his continuous decision to stay on Suðuroy when many others left (Pedersen & Zittoun, 2022). People’s lives are intimately linked to others, which some refer to as *linked lives* (Elder & Giele, 2009), providing social recognition (Zittoun & de Saint-Laurent, 2015). The weight of these affective relations lends the imagination power.

Einar’s affective ties to his two brothers buoyed his imagination of the future, constituting an immovable point through crises:

I actually have no explanation of why I’m so happy about being here, ever since I was a child […] I actually have no other explanation. I was doing well all the time, and all three brothers, we are the example, and there are a lot like me, that is, the inhabitants here. It was nothing like that we had—our way of living was good enough. It wasn’t any kind of luxury. I wouldn’t say that at all, but we were doing good, and we grew strong and all healthy, and we had no daily problems despite our means being small. But it was like that everywhere, and perhaps there had been some longing for going abroad—they had thought that “we must be able to get it better than we have it here”. That was probably part of the problem—that people wanted to go somewhere else—but when you are happy about staying here and have had a fine and happy upbringing overall. Perhaps I can say that my mother was a lot—she did not think too far ahead; she took everything one day at a time and was not worried about anything. And this made life so much easier. If she had walked around with a heavy mind, as many Faroese had back then, then we would say: “We need to get away fast”. I believe. But I experienced Suðuroy when it was on 6,000 people—just before the war, under the war, and after the war—we were just about 6,000 people. But our condition, we simply had it so well, and we three brothers were so connected that we formed a unit in some way.

Einar described the bond between him and his brothers as being akin to that of a “small unit”. Although they did not grow up in “luxury”, none of them had complaints nor expressed enduring longings to go abroad. They literally shared the same bedroom until they were married away one-by-one, as Einar jokingly added, and stuck together throughout life. Their affective relation delegitimised imaginations of abandoning ship when crises hit and therefore engendered staying. Leaving his brothers was unimaginable for Einar. He naturally felt sad whenever the fishery industry took one of its inevitable dives, but his tight-knit family unit gave comfort because, regardless of what happened, he had his brothers. This affective tie sedentarised his imagination of the future despite the uncertainty produced by crises.

Einar’s affective tie to his mother also shaped his imagination of the future. He repeatedly recalled how his mother did not worry much about the future. Rather, she lived on a day-to-day basis and did not take life too seriously. Learning from his mother, Einar described himself as never inclined to the heavy mindedness that many of his peers succumbed to, instead developing a presentist outlook. Hardship did not consume Einar. He focused on the tasks before him. His mother therefore functioned as a role model for both imagining and engaging with the future.

While temporalities and experiences shaped Einar’s imagination of the future amidst crises, the affective relations constituted an immovable point and instilled a specific engagement with the future, thus becoming a mitigating factor. On the one hand, Einar’s affective ties to his brothers contributed to a feeling of comfort and the certainty that they would be around regardless of what might happen. On the other hand, Einar’s presentism, socially recognised by his mother, centred his imagination on the immediate future. The longer-term future remained largely outside the temporal horizon. When societal crises hit, his affective ties and orientation towards the immediate future aided the imagination. Contrary to studies mentioned earlier (Griffiths, 2013, 2014), Einar’s presentism appears less a result of being in a state of either chronic or temporary crisis than of past crises being used as resources for present ones. Crises did not per default disrupt the temporalities; rather, they were an integral feature of life, and he imagined a brighter future after the storm.

## Living and navigating societal crises

After having identified the temporal and affective factors supporting Einar’s ability to imagine a future through societal crises, I return to his descriptions of living through periods commonly defined as such. These were obviously unpleasant for Einar, and he felt sad for many reasons—sad that people lost their livelihoods, sad that many people left the island, sad that the village emptied, sad that infrastructures deteriorated, and sad that people slowly started to lose interest in fishery altogether:

Einar: It was sad. Honestly. I have been part of the upswings, high conjectures, several times, and several downswings, but when the fishing fleet began to shrink and, how do you say, there was not so many people who were interested in fishery, because they could earn their money elsewhere, it was a little difficult to witness.

Oliver: When was this about?

[…]

Einar: I would say, let me, if we should start… the 50s were a little difficult. There was so much striking, and they did not earn much money because there was so much striking. We got some downswings in the 50s, and then another upswing when they had started to develop a line fishery fleet and modernised it. They had some big line fishery ships built in Norway and some in France. Then it started to look brighter as the ships started to sail on the fjord again. It went up again, and then in the 70s there were oil problems. The oil had become too expensive, and the fishery could almost not stay solvent with the modern motorised ships. Then we got a massive down-swinging conjecture. Then it went up a little again, and the fleet did not get much bigger, but it was transformed. Away from the sloops that slowly disappeared. They had become old and had served their time—and [the newer ships] did not require as much of a big crew. Then more and more people started to find their source of income many other places, and there were naturally some trawlers starting to enter the fishery […]. But, as one says, there was still development of the fishery, though the catch was sailed directly abroad without arriving on solid ground in the Faroes.

Einar lived through economic highs and lows, applying the technical term *conjecture* (direct translation from Danish), which captures the business cycles intrinsic to neoliberal economics. He identified several major downswings that were also labelled crises in wider society as well. Rather than occurring suddenly, they were gradual and caused by a mix of local and global factors. The first occurred in the early 1950s. The Second World War provided an unlikely but lucrative opportunity for the Faroese fishery fleet, which supplied fresh fish to the British Isles because British ships were appropriated for the war effort. While transporting fish was a perilous endeavour, it also facilitated an unprecedented accumulation of wealth for Faroese society. Most Faroese ships of the era were made of wood, and many had sailed the seas since the around the beginning of the 19^th^ century. Therefore, the capital amassed was used to acquire second-hand steel trawlers. However, these ships struggled to be competitive internationally because of inflated prices and a relentless demand for repairs (Wylie, 1987). The reintroduction of import taxes in Britain and the rising price of coal exacerbated the Faroese fishery fleet’s struggle to remain solvent (Justinussen, 1997). The tides turned when new financing schemes was introduced to reconstruct the fishing fleet and the first processing plants on Faroese soil were constructed in the mid-1950s. This signalled the end of the crisis. Indeed, witnessing the emerging infrastructure and the increasing mobility of ships on the fjord epitomised that, for Einar, progress had arrived, and the future had returned. With the official introduction of Faroese home-rule in 1949 (Debes, 2001; Sølvará, 2020), Einar began to work as a customs officer, which meant his situation remained less precarious than that of others and naturally influenced his experience of the crisis (Rydstrom, 2022).

Einar identified the next substantial societal crisis as transpiring in the 1970s. Rocketing oil prices created devasting reverberations throughout the global economy, causing the now motorised Faroese fishing fleet to struggle with profitability. However, once again, new financing schemes boosted modernisation. Alongside the stabilisation of oil prices, such programmes kept the Faroese fleet afloat but decreased the number of jobs available in the industry. Einar’s imagination of the future was not obliterated despite both these crises leading to spikes in material degradation and people leaving, which produced increases in emptying.

The emptying remained at a manageable level in the first two societal crises, and therefore rebuilding was still imagined as the promised outcome. It was a question of waiting. Crises were inevitable but also imagined as temporary events. Hence, existing temporalities and experiences with previous crises permitted Einar to retain an imagination of the future. The Janus-faced fishery industry fashioned crises and simultaneously served as the condition for eventual improvement. Einar’s interest in local histories further supported the understanding that crises were indeed followed by an upswing in the form of new fishing ships and infrastructural improvements. Past crises would again become a resource for present ones. Then came the 1990s.

I want to say that when we entered the conjectures in the 1990s, then it was time for a downswing again. I was very sceptical whether we had enough energy, people, and initiative to build it all up again, so that we got on the same level as the others [villages]. At the same time, they had built a lot up in Torshavn and Klaksvik, and especially Klaksvik was the strong city regarding the fishery industry, and Torshavn was also following along quite well. Though, Torshavn was also a bigger city due to the organisation, or administration as it is called. We slowly climbed up, but not good enough […] from the 90s and onwards, I had a very bleak outlook. We just went down and down. I was very sad back then, but I thought: “I’m getting old, so I’ll stay here—I won’t move”.

Oliver: So, there wasn’t a time when you thought: “Now it looks so bleak that I will move”?

Einar: No, not from the island. The conjectures were about the same all around the Faroe Islands, but we were—because we had been so strong, it also took a little harder on us. We had been on the high points.

The societal crisis in the 1990s was the result of multiple culminating factors that produced an overheated economy. The collapse was caused by the expansion of the fishing grounds to 200 nautical miles, depleting fishing stocks, localising policies, declining prices, and the establishment of the Raw Fish Fund to artificially keep the price fixed (Debes, 2001; Holm & Mortensen, 2002; Justinussen, 1997). Einar’s description of the 1990s downswing differed noticeably from the previous two crises in terms of both the frequency and manner in which he spoke of it. The consequences became so locally widespread, tangible, and clearly visible that they were hard to ignore, challenging his future. The 1990s crisis gained a more personal character due to its intensity (Rydstrom, 2019) and local manifestations, with banks collapsing, unemployment reaching 20%, and up to 10% of the population leaving (Guttesen, 1996), particularly the younger segment (Holm & Mortensen, 2002). Moreover, the synchronic and diachronic contrast between how life on the island once was and how it proceeded elsewhere accentuated the experience of crisis. Crises entangle and are relative. Tvøroyri was once a thriving and lively village, but houses were left abandoned in the 1990s. Experiencing such a direct form of emptying marred Einar’s belief in the self-correcting mechanism intrinsic to temporalities and made focusing on the present nonsensical. With so many people having left, he doubted whether sufficient initiative remained to allow recovery. The crises had broken the temporal seams This resonates with other studies in which people seemed to lose hope in the return of a promised future and moved in search of it elsewhere or became trapped between what was lost and what is not yet visible (Dzenovska, 2018).

Einar unwavering imagination of the future diminished in tandem with the flight of people, capital, and initiative. What had maintained the temporality seemed lost (Adam, 1995). The emptying challenged the very foundation of the future because Einar doubted whether the fishery industry could rebound. Deteriorating material conditions, disappearing social events, empty houses, and high unemployment stood in stark contrast to Einar’s memory of fishery’s heydays. Back then, more than 6,000 people lived on the island (Holm, 2007). The circumstances reached a point at which Einar had difficulties imagining the future.

This crisis was largely confined to the Faroe Islands and hit Suðuroy particularly hard due to its overdependence on fishery. Einar’s doubts tarnished his ironclad decision to stay, and he briefly considered moving away, which is emblematic of the more personal nature of this crisis. The promised upswing was momentarily obscure, and the event threatened to become chronic (Vigh, 2022). Focusing on the present while waiting out crisis became untenable. However, Einar eventually decided to stay due to his age, his brothers still living on the island, his stable socioeconomic position, and his attachment to the village and its inhabitants. Here the affective ties, as well as Einar’s circumstances, mitigated the downturn’s adverse effects and compensated for the breakdown of his presentist imagination and the disruption of temporality.

When I asked Einar why he chose to stay during the 1990s crisis, he never mentioned going abroad to live. Instead, he replied that the situation was similar in the rest of Faroes. He also highlighted how Suðuroy’s historical role as one of the Faroes’ socioeconomic and technological developmental centres amplified the experience of crisis. The heydays of fishery on Suðuroy functioned as a baseline for his imagination, highlighting the relational aspect of crises. Einar worried that Trongisvágur (one of the four villages that now fall under Tvøroyri) would “die”:

Oliver: What about the 1990s?

Einar: No, it had reached the point where, do I dare say today, had it not been for the pelagic history and the people from Gøta, then Trongisvágur would probably have died, so it had been a small village with people who could not really do much. I almost believe so. I have said, and I also believe that in terms of time, even though we were many people. We had dropped all the way below 1,300 people at some point.

Oliver: That is not a lot.

Einar: It might not tell you much, but from 1,700, there were 400 Faroese less in a village like this and the initiative was—there was way too little initiative among people.

The future was not only temporarily uncertain but had also began to be imagined as undesirable, with Einar imagining one of the villages might “die”. Einar experienced the consequences of the crisis to be so dire that he imagined Trongisvágur might have emptied completely without external intervention (a fishery company from the northern Faroes decided to build a factory in Tvøroyri). Few people would have been left in the village and social life would have died. The collapse of the fishery industry and the subsequent restructuring challenged the imagination of the future Einar used to retain throughout societal crises. It is almost as if he feared that the circumstances might turn chronic. The normalised yet exceptional states of crisis inherent to fishing threatened to become a permanent condition, challenging Einar’s ability to use past crises as resources for the present. This is likely due to the 90s crisis’ intensity and scope. Indeed, the crisis adopted a more personal character, and he questioned whether to leave or not. Certainty regarding the long-term future could no longer be taken for granted. However, Einar’s imagination of the future was ultimately restored in correspondence with the return of the Faroese fishing fleet due to its, as he phrases it, unprecedented adaptability. He is no longer worried.

## Concluding remarks

In this article, I followed Einar’s imaginations of the future through three societal crises. I explained how temporalities, previous experiences, presentism, stable socioeconomic circumstances, and affective relations supported his imagination of the future amidst societal crises. Experiences of past crises were used to make sense of present ones and to guide his imagination, creating a form of temporal resonance between crises. Einar’s imagination of the future was simultaneously characterised by certainty and uncertainty, promising blue skies after the storm, with the near future being primary. Einar imagined crises as naturally occurring, temporary, and always followed by “progress”. The storm just needed to be weathered by focusing on the present. However, the magnitude and intensity of the 1990s crisis and the slow process of emptying made Einar question whether enough resources, people, and initiative remained to recover. While briefly contemplating leaving the island, as did many others between 1989 and 1994, he decided to stay. The future lost its certainty, and the widespread emptying made focusing on the present untenable. The 1990s crisis became personal for Einar because it threatened to become a chronic state, whereas previous crises were considered temporary in nature and therefore not destructive of the future. However, due to his age, affective ties to the people and the place, and his socioeconomic position at the time, Einar stayed on Suðuroy. This point demonstrates that there is no universal experience of crises. Instead, what constitutes crises and how they manifest will vary according to people’s trajectories and circumstances. For example, in Einar’s case, past crises were largely productive. However, for others, previous crises might amplify current ones. It is therefore important to reject an idealised approach to how crises interact over time.

Einar’s story underscores that crises can be entangled diachronically and synchronically and can constitute more than merely unprecedented events. His story also cautions against defining crises from the “outside”. Einar’s experiences of crises were neither chronic nor temporary states of emergency; nor were his imaginations of the future necessarily uncertain. Each crisis has unique temporalities and intensities (Rydstrom, 2022). Einar described crises as forms of normalised yet temporary states of exception; they were imaginable but still, to varying degrees, disruptive of social and economic life. Crises were part of the temporalities, and his imagination was therefore neither blocked nor flourishing; rather, it remained abstract and hopeful. This resembles what Hage (2009) calls “waiting out”. However, the 1990s crises threatened to become chronic, with “progress” no longer appearing certain due to the crisis’ magnitude (Bergman-Rosamond et al., 2022). Echoing others arguing for the integration of first- and third-person perspectives (Zittoun et al., 2023), I argue it is necessary to understand when people encounter crises, how their unique trajectories and experiences of crises come into play, and what form the future takes. Calling something a crisis is an epistemological claim (Roitman, 2014) but can overlook when and how crises manifest for people—if at all. Furthermore, crises do not merely have their own unique temporalities, their temporalities are entangled with pre-existing ones. To explore when something becomes a crisis, it is essential to understand people’s experiences, their experience of time, and their imagination within a local context.

Additionally, I have attempted to demonstrate that societal crises cannot be assumed to either impoverish or nourish people’s imaginations. Crises can be used as a resource for the imagination, explaining why “waiting out” was a viable strategy for Einar. Rather than focusing solely on people’s experiences and imaginations during single crisis, it is important to also account for the interplay between crises and the temporalities that exist prior. Possibly, a positive resolution of a previous crises during the life course might create a temporal resonance, reinforcing that things will get better. Past crisis experiences might even serve as a blueprint later in life. However, with the caveat that the opposite might also hold true, past crises can also amplify the experience of current ones. This extends to the entanglement of crises often discussed in anthropology across time and takes individual experiences seriously (Fassin & Honneth, 2022). To account for the socio-material and global-local dynamics, I integrated the concept of emptiness (Dzenovska, 2020). Doing so captures the link to disappearing infrastructures, the practices that emerge, and how people imagine under circumstances involving either a sudden or slow crisis. The flight of people, empty streets, and abandoned houses appeared to drive Einar’s trepidation that one village might “die”, reducing the possibilities for imagination and action (Glăveanu, 2020). Beyond theoretical attempts (Zittoun et al., 2020), the material grounding of imagination and its linkages to socioeconomic and political changes have been somewhat lost given the focus on semiotics often exhibited in sociocultural psychology’s literature concerning the imagination. (Pedersen, 2022). Moreover, studying the manifestations of crises might represent a further step to embed the process of the imagination in material conditions, connections, power relations, and diverse temporalities.

Sociocultural psychological research, especially concerning imagination, can gain from a closer exploration of temporalities and crises. Current understanding of what defines a crisis, its conditions, and its relations is still embryonic and by emphasising people’s experiences, sociocultural psychology can advance this strand of research. An important factor determining whether a societal crisis becomes a personal might be the ability to imagine the future. Echoing Scheper-Hughes, this provides a cautionary tale against studying crises solely as exceptional states or commonly assumed transitions. While such moments offer a natural approach to explore development and transformation (Zittoun, 2009), it might also side-line other dynamics. This observation brings to the fore the idea of studying relationship between crises, both societally and over the life course. Previous crises can become resources for navigating and making sense of present crises. To discern when or whether this happens, it is crucial to consider life courses and experiences that can both mitigate or amplify the impact on societal crises. As such, research needs to incorporate diachronic and synchronic dimensions to account for how individual’s life course and imagination unfold in times of crises. Here sociocultural psychology is particularly apt. Research should also be careful not to assume that the future was either lost or uncertain during crises. By focusing on the experience of crises, researchers can better nuanced and unpack the role of temporalities and what a crisis means, to whom, and how it impacts the future. This approach goes beyond merely observing how various crises intersect in the present. It calls for an awareness of how past crises inform and shape the current and future ones.

While time and space are interwoven (Marková & Novaes, 2020; Valsiner, 2014), much of sociocultural psychological research does not harness its own potential. It is broadly concerned with the link between the sociocultural environment and the person, and here it is imperative to include different temporalities as well. As Adam (1995) contends, this recognition is not about abandoning irreversibility but to also account for other experiences of time and the factors that shape them. This proposition can impact life course research (Neale, 2021), which often studies how lives unfold in irreversible time. However, adopting a more social and experiential definition provides phenomenological insights on when ruptures and transitions happen, such as when people feel existentially stuck (Hage, 2009). If the objective is to study human development over time and the significance they attribute to their life course at different stages, much is still left to be done by attuning to temporalities. Exploring temporality in sociocultural psychology demands attention to how people’s sense of time is constructed—s linking it firmly to space and sociocultural environment—and tracing how temporalities might impact developmental processes.

## Note

1.
https://nccr-onthemove.ch/projects/small-localities-at-the-outskirts-of-europe-transnational-mobilities-diversification-and-multi-scalar-place-making/
